# Sterile Punktion großer Gelenke

**DOI:** 10.1007/s00064-022-00786-3

**Published:** 2023-01-17

**Authors:** Viktor Labmayr, Franz Josef Eckhart, Maria Smolle, Sebastian Klim, Stefan Franz Fischerauer, Gerwin Bernhardt, Franz Josef Seibert

**Affiliations:** 1 Universitätsklinik für Orthopädie und Traumatologie, Medizinische Universität Graz, LKH-Univ. Klinikum Graz, Auenbruggerplatz 5, 8036 Graz, Österreich; 2 AUVA Unfallkrankenhaus Steiermark, Standort Graz, Graz, Österreich

**Keywords:** Infektion, Gelenkspunktion, Erregernachweis, Intraartikuläre Injektion, Gelenkerguss, Infection, Joint puncture, Pathogen testing, Intraarticular injection, Joint effusion

## Abstract

**Ziel:**

Die Punktion großer Gelenke erfolgt einerseits zur Diagnostik und andererseits zur Behandlung von Gelenkspathologien. Mittels Punktion kann eine rasche Schmerzreduktion durch Entlastung von Ergüssen oder intraartikulären Hämatomen erfolgen. Das gewonnene Punktat erlaubt schon bei der Beschau mit dem freien Auge eine erste Einschätzung und in der Folge eine mikroskopisch-zytologische sowie mikrobiologische Befundung im Labor.

**Indikationen:**

Die Punktion eines großen Gelenkes ist zur Diagnose und/oder Therapie bei entzündlichen, traumatischen oder postoperativen Gelenksveränderungen angezeigt. Diagnostische Punktionen dienen der Punktatgewinnung, der differentialdiagnostischen Schmerzausschaltung oder (selten) der Kontrastmittelapplikation für die Magnetresonanzarthrographie. Therapeutische Punktionen ermöglichen die Injektion von Medikamenten oder plättchenreichem Plasma (PRP) sowie die Entlastung bzw. Drainage von Ergüssen.

**Kontraindikationen:**

Bei entzündlichen und insbesondere eitrigen Hautveränderungen im Punktionsbereich sind Gelenkpunktionen kontraindiziert.

Obwohl keine absolute Kontraindikation, ist bei Einnahme blutgerinnungswirksamer Substanzen Vorsicht geboten.

**Operationstechnik:**

Besonderes Augenmerk ist auf ein steriles Vorgehen zu legen. Unnötige Schmerzen können durch das sterile Setzen einer Lokalanästhesiequaddel, „sichere“ Punktionspunkte sowie vorsichtiges Hantieren mit den Punktionskanülen vermieden werden.

**Weiterbehandlung:**

Punktate müssen entsprechend den lokalen, intrahospitalen Richtlinien zeitgerecht aufgearbeitet bzw. entsorgt werden. Die Punktionsstellen werden mit sterilem Pflasterverband abgedeckt, bei Medikamentenapplikation die Gelenke zur Verteilung des Wirkstoffes passiv durchbewegt. Danach erfolgt eine Kompressionsbehandlung mit elastischer Bandage von distal nach proximal bis über die Punktionsstelle zur Vermeidung von Nachblutungen oder sofortiger Ergussneubildung.

**Fakten:**

Werden sämtliche Maßnahmen der Sterilität bei der Punktion großer Gelenke eingehalten, ist davon auszugehen, dass Infektionen mit 0,04–0,08 % (4 bis 8/10.000 Punktionen) nur sehr selten auftreten. Das Risiko für falsch positive Erregernachweise ist äußerst gering.

## Abstract (en)

**Objective:**

Puncture of large joints is performed for diagnostic purposes on the one hand and for the treatment of joint pathologies on the other. Puncture can be used for rapid pain relief by relieving effusions or intra-articular hematomas. The obtained puncture specimen allows immediate visual assessment and subsequent microscopic-cytological and microbiological evaluation in the laboratory.

**Indications:**

The indication for puncture of a large joint is for diagnosis and/or therapy of inflammatory, traumatic or postoperative joint problems. Diagnostic punctures are used to obtain punctate, to differentiate the location of pain or (rarely) to apply contrast medium for magnetic resonance arthrography. Therapeutic punctures allow the injection of drugs or platelet-rich plasma (PRP) as well as the relief or drainage of effusions.

**Contraindications:**

If there are inflammatory skin alterations—especially purulent inflammation—joint punctures through these lesions are absolutely contraindicated. Special attention is necessary if the patients are on anticoagulants.

**Surgical technique:**

Absolute sterile handling is mandatory. Unnecessary pain can be avoided by a sterile skin wheal of local anesthesia, safe puncture points, and careful handling of the cannulas.

**Postoperative management:**

Joint aspiration material has to be handled according to the local, intrahospital rules in a timely manner. Puncture sites are covered with sterile dressings, and if intra-articular medication is administered, the joints have to be passively moved through the range of motion to distribute the medication. Thereafter, compression therapy from distally to proximally while also covering the puncture site avoids recurrence of swelling or hematoma.

**Facts:**

If sterile conditions are guaranteed, infections rarely occur (0.04–0.08%, 4–8/10,000 cases). The risk of false-positive detection of microorganisms is extremely low.

## Lernziele

Nach der Lektüre dieses Beitrags …

- sind Ihnen die Indikationen und Kontraindikationen zur Punktion großer Gelenke bekannt,
- kennen Sie die technischen/anatomischen Voraussetzungen und Bedingungen zur sterilen Punktion von großen Gelenken und deren Komplikationen,
- können Sie Patienten „state of the art“ aufklären und über Risiken und Komplikationen informieren,
- sind Sie in der Lage, die Punktate zu interpretieren und entsprechende Konsequenzen zur weiteren Untersuchung und Behandlung zu ziehen.

## Vorbemerkungen

In Orthopädie und Unfallchirurgie sind **Gelenkpunktionen**

Gelenkpunktionen

tägliche Praxis. In der Unfallchirurgie werden Gelenke bei ausgeprägtem Hämarthros nach Unfall punktiert und entlastet. In der Orthopädie werden akute und chronische Gelenkergüsse punktiert mit dem Ziel der Diagnostik und schnellstmöglichen Therapieeinleitung. Bei septischen Prozessen und Ergüssen der Gelenke besteht eine dringliche Indikation zur Punktion und Aspiration von Gelenkflüssigkeit.

Vor jeder Punktion sollten die spezifischen anatomischen Gegebenheiten des Gelenks gegenwärtig sein, um Schmerzen und Verletzungen zu minimieren.

Eine Gelenkpunktion, auch **Arthrozentese**

Arthrozentese

genannt, kann aus **diagnostischer Indikation**

diagnostischer Indikation,

z. B. Abklärung der Ätiologie einer akuten Arthritis, oder aus **therapeutischer Indikation**

therapeutischer Indikation,

z. B. zur Schmerzlinderung, Drainage eines Ergusses oder Injektion von Medikamenten, erfolgen.

Bei allen Punktionen und Injektionen der Gelenke wird die Induktion eines **Gelenkinfekts**

Gelenkinfekts

gefürchtet. Bei Patienten mit verminderter Abwehrlage wie Behandlung mit immunsuppressiven Medikamenten, bei vorausgegangener Infektion in diesem Gelenk und bei Stoffwechselkrankheiten, insbesondere beim Diabetes mellitus besteht ein erhöhtes Infektrisiko [1, 2]. Bei diesen Patienten ist Zurückhaltung v. a. in der Anwendung von Kortikoidinjektionen geboten, weil dadurch die lokalen entzündungshemmenden Mechanismen gebremst werden [1, 2].

Es werden folglich diagnostische und therapeutische Punktionen und dringliche bzw. notfallmäßige Punktionen unterschieden, wobei es hier zu fließenden Übergängen kommt. Bei der Punktion eines chronisch gereizten Gelenks mit ballonierter Kapsel kann bereits makroskopisch eine Erstbeurteilung des Punktats erfolgen. Danach kann es weiter mikroskopisch, zytologisch und bakteriologisch untersucht werden. Die Entlastung des Gelenks reduziert die Schmerzen und kann die Beweglichkeit verbessern.

Die Indikation zur **dringlichen Punktion**

dringlichen Punktion

besteht bei fiebrigem Patienten mit einem überwärmten, geschwollenen und geröteten Gelenk. Dies gilt sowohl für das native Gelenk als auch für das voroperierte Gelenk (z. B. nach einer Arthroskopie oder bei liegendem Gelenkersatz). Im diagnostischen Algorithmus muss schnell entschieden werden, ob ein septisches (d. h. bakterielles) oder aseptisches Ereignis vorliegt. Im Falle einer septischen Entzündung sind umgehend weitere chirurgische Schritte erforderlich.

Bei frischem Gelenktrauma können sogenannte „Fettaugen“ im Gelenkpunktat Hinweis auf eine Verletzung der osteochondralen Grenzschicht geben, wie z. B. bei okkulten Gelenkfrakturen mit osteochondraler Abscherung oder bei undislozierten Tibiaplateaufrakturen. Die Fettaugen resultieren aus dem Übertritt des lipidhaltigen Knochenmarks ins Gelenk. Weitere Abklärung mittels Computertomographie (CT) oder Magnetresonanztomographie (MRT) ist üblicherweise angezeigt.

Die Punktion steriler Gelenke kann auch dazu genutzt werden, **Medikamente**

Medikamente

in den Gelenkraum zu injizieren, darunter Kortison zur Linderung aseptischer Entzündungszustände, ein Lokalanästhetikum zur Behandlung akuter Schmerzzustände und Hyaluronsäure zur Behandlung von Chondropathien.

## Prinzip der Gelenkpunktion

Bei der Punktion großer Gelenke wird eine Nadel oder ein Trokar – nach Stichinzision – in das Gelenkkavum geschoben. Mit Hilfe einer angesetzten Spritze wird Gelenkflüssigkeit aspiriert oder ein Medikament in die Gelenkhöhle appliziert. Einer der wichtigsten Aspekte dieser Maßnahme liegt darin, keine Keime ins Gelenk zu verschleppen. Der Eingriff soll möglichst schmerzfrei und ohne iatrogene Verletzungen ablaufen. Die meisten Gelenkpunktionen werden in der Klinikambulanz oder der Praxis durchgeführt und nicht in einem Operationssaal, dennoch gelten die gleichen **Prinzipien der Hygiene**

Prinzipien der Hygiene.

Punktionsbedingte Infekte müssen vermieden werden [3, 4]. Die von vielen nach wie vor präferierte und angewandte No-Touch-Technik sollte heute durch **sterile Techniken**

sterile Techniken

ersetzt sein [5].

Vor einer Punktion sollte nach ausreichender **orientierender Palpation**

orientierender Palpation

des Gelenks bei guten anatomischen Kenntnissen Klarheit über die **Punktionsstelle**

Punktionsstelle

herrschen. Hilfreich ist die Markierung der Punktionstelle auf der Haut. Ein Markierung mit dem Stift wird allerdings durch das anschließende sterile Waschen verblassen oder verschwinden. Hier kann ein einfacher Trick angewandt werden: Der Untersucher drückt mit seinem (sauberen) Fingernagel ein Kreuz auf die Haut des Patienten. Die dadurch entstandene Eindrückung der Haut übersteht das sterile Waschen und ist wenige Minuten danach noch gut erkennbar. Alternativ kann ein Kugelschreiber (ohne Mine) verwendet werden. Eine adäquate Lagerung ermöglicht eine entspannte Muskelsituation, die für eine schmerzarme Arthrozentese entscheidend ist.

Im Folgenden sollen die Ziele, Indikationen, technischen Voraussetzungen, die sterile Durchführung als auch die ggf. nötige Aufarbeitung des Punktats dargestellt werden, um eine bestmögliche Sicherheit und einen bestmöglichen Behandlungsbenefit für den Patienten sicherzustellen [6, 7]. Ebenso werden alle großen Gelenke angesprochen, im Speziellen Schulter, Ellenbogen, Handgelenk, Hüftgelenk, Knie, Sprunggelenk mit ihren jeweiligen Besonderheiten und zusätzlich das Iliosakralgelenk (ISG).

## Prinzip der Maßnahme

Bei der Punktion sollte der **kürzeste Weg zum Gelenk**

kürzeste Weg zum Gelenk

gewählt werden. Eine Ausnahme stellen entzündlich veränderte Hautareale oder Tumoren dar. Die Nadel sollte so klein wie möglich, aber so groß (und lang) wie nötig gewählt werden. Bei einer Punktion mit erwartetem dickem Sekret kann eine stärkere Nadel von Vorteil sein, hingegen wird man bei reiner Infiltration eine dünnere Nadel wählen. Vor allem bei Patienten mit erhöhtem Blutungsrisiko sind dünnere Nadeln von Vorteil, die zudem schmerzärmer sind.

Die Punktion größerer und gut zugänglicher Gelenke (z. B. Knie) erfolgt meist **landmarkengestützt**

landmarkengestützt

**Figure 1:**
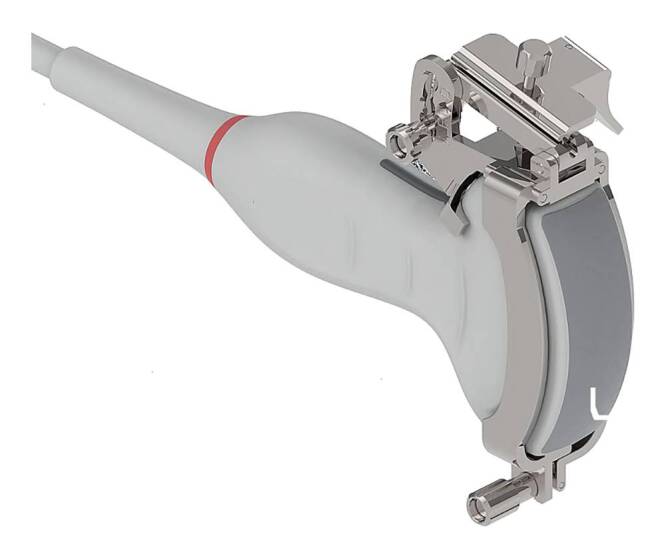

[6, 7, 8]. Bei schlechter zugänglichen Gelenken (insbesondere Hüfte, Schulter) wird die Punktion **ultraschallgestützt**

ultraschallgestützt

erleichtert [6, 8, 9, 10]. Es hat sich gezeigt, dass im Vergleich zum landmarkengestützten Verfahren die ultraschallgeführte Punktion schmerzärmer und mit besserem Punktionserfolg durchgeführt werden kann ([11, 6, 7]; Abb. 1). Die Bildwandler-gestützte Punktion ist bei einigen Gelenken wie dem Hüftgelenk und Handgelenk hilfreich. CT- oder MRT-gestützte Punktionen sind möglich, werden in dieser Arbeit nicht erläutert, da sie Spezialindikationen vorbehalten sind [12].

### Vorteile

- Minimal-invasive, risikoarme Intervention [13]
- Schnelle Diagnostik (primär: Punktatfärbung/-viskosität; sekundär: Synoviaanalyse mit Mikroskopie, Zellzahlbestimmung und Antibiogramm) [6, 7, 14]
- Rascher Therapieerfolg (z. B. Entlastung eines Reizergusses mit/ohne Injektion von Kortison bei chronischer Synovitis) [6, 7]
- Einfach zu erlernende Technik

### Nachteile

- „Punctio sicca“ (trockene Punktion) möglich
- Schmerz-, Hämatom- und Infektrisiko bei inkorrekt durchgeführter Punktion [13]
- Erfolgsrate von Erfahrungswert abhängig [15, 16]

## Indikationen

1. Diagnostisch
  Keimgewinnung bei Verdacht auf septische Arthritis [6, 7]
  Nachweis von Kristallarthropathien (z. B. negativ doppelbrechende Uratkristalle, schwach positiv brechende Calciumpyrophosphatkristalle)
  Frakturnachweis (Liphämarthros = Fettaugen) [17]
  Ausschluss von gelenkpenetrierenden äußeren Verletzungen (intraartikuläre Injektion von Kochsalzlösung gemischt mit Methylenblau; kutaner Austritt des Farbstoffs zeigt Verletzung der Gelenkkapsel an) [18, 19, 20]
2. Therapeutisch
  Hämatomentlastung (z. B. Radiusköpfchenfraktur) [21]
  Abpunktion von Gelenkerguss (z. B. degenerativer Reizerguss, Synovitis)
  Injektion von Medikamenten (z. B. Hyaluronsäure, Kortison, Platelet-Rich-Plasma [PRP]) [22]

## Kontraindikationen

- Entzündungen und andere Hautveränderungen im Bereich des zu punktierenden Gelenks (z. B. großflächige Exkoriation, Rötung, Erysipel) [6, 7]

### Absolute Kontraindikationen

- Schwere Gerinnungsstörung (Optimierung bzw. Korrektur vor der Punktion erwägen)
- Bakteriämie (z. B. *Staphylococcus-aureus*-Bakteriämie; bei therapeutischer Punktion zur Entlastung eines septischen Gelenks als relativ einzustufen)
- Zeitnahe geplanter endoprothetischer Gelenkersatz (innerhalb von 6 Monaten; Gefahr der Keimverschleppung nach intraartikulär bei unsachgemäßer Durchführung der Punktion, mit nachfolgender Besiedelung des Implantats durch Biofilm-formende Bakterien) [23]
- Endoprothese im zu punktierenden Gelenk (Risiko für iatrogenen Protheseninfekt bei unsachgemäß durchgeführter Gelenkpunktion mit Keimverschleppung nach intraartikulär; bei Verdacht auf Vorliegen eines Protheseninfekts als relativ einzustufen) [6, 7]
- Einnahme von Blutverdünnern wie z. B. direkten oralen Antikoagulantien (DOAK; wie z. B. Apixaban, Rivaroxaban) oder auch Vitamin-K-Antagonisten. Es besteht die gering erhöhte Gefahr für Nachblutungen bzw. Hämarhtrosbildung [1, 9, 24, 25]
- Schlecht eingestellter Diabetes mellitus (relativ; zuerst Alternativen zur Gelenkpunktion in Erwägung ziehen) [6]

### Relative Kontraindikationen

- Hautläsion an der idealen Punktionsstelle (alternative Punktionsstelle suchen)
- Bekannte Bakteriämie
- Benachbarte Osteomyelitis
- Unkontrollierte Koagulopathie
- Gelenkprothese
- Immunsuppression (relativ; zuerst Alternativen zur Gelenkpunktion in Erwägung ziehen) [26, 27]
- Wiederholte Gelenkpunktionen (relativ, bei therapeutischer Punktion) [26, 27]

Die prophylaktische oder therapeutische Antikoagulation sowie eine rheumatologische Grunderkrankung stellen keine Kontraindikation für die Gelenkpunktion dar [26, 27].

## Komplikationen

### Komplikationsvermeidung bei Punktion

**Steriles Arbeiten**

Steriles Arbeiten

ist zwingend. Die intraartikuläre Punktion wird meist zur Risikogruppe 3 gezählt [3, 4].

- Ausreichend lange und genügende Desinfektion im und um das Punktionsgebiet nach Standard [3, 4]
- Sterile Abdeckung durch z. B. Lochtuch [3, 4]
- Sterile Handschuhe [3, 4]
- Mund-Nasen-Maske [3, 4]
- Stichinzision mit dem Skalpell, um Transfer eines Gewebekerns aus dem Nadelinnenraum bei großlumigen Kanülen zu verhindern (optional) [28]; tierexperimentell konnte gezeigt werden, dass dies von der Nadelgröße, aber auch von infiziertem Gewebe an der Punktionsstelle beeinflusst wird [29].
- Forcierte Kanüleneinbringung kann zu Knorpelabrasionen und -schaden führen. Der Vorschub ist zu stoppen, sobald Flüssigkeit über die Nadel austritt, womit Knorpelschäden vermieden werden.

### Komplikationsvermeidung bei Injektionen

- Medikamente in Einzeldosisflaschen
- Desinfektion der Oberseite des Fläschchens
- Wechsel der Nadel (stumpfe Aufziehkanüle) nach Aufziehen des Medikaments
- Direkte zeitnahe Verwendung der Spritze – nicht lange vorher aufziehen

## Patientenaufklärung

### Aufklärung Punktion von Gelenken

Eine Gelenkpunktion oder medizinisch auch Arthrozentese genannt ist eine ärztliche Intervention, der eine klare **Nutzen-Risiko-Abschätzung**

Nutzen-Risiko-Abschätzung

vorausgeht. Obwohl selten, so besteht dennoch die Gefahr verschiedener Nebenwirkungen und Komplikationen, über die der Patient informiert werden muss.

Die Einhaltung absoluter Sterilität sowohl der Punktionsstelle als auch des Instrumentariums ist unerlässlich. **Asepsis**

Asepsis

hat hierbei den höchsten Stellenwert, da die iatrogene Infektion eines Gelenks während der Punktion eine ernst zu nehmende und gefürchtete Komplikation darstellt. Eine Risikoabwägung muss deshalb mit allen Patienten gemeinsam erfolgen [1].

Im Rahmen des Aufklärungsgesprächs sollte eine **Anamnese**

Anamnese

bezüglich gerinnungshemmender Medikation sowie einer evtl. vorhandenen Erkrankung, welche die **Blutgerinnung**

Blutgerinnung

beeinflussen könnte, durchgeführt werden. Bei Vorliegen einer solchen Erkrankung oder bei Einnahme von Gerinnungshemmern ist die Indikation (relative Indikation wie oben erwähnt) zur Punktion streng zu stellen, da sich dadurch das Risiko zur Ausbildung eines Hämarthros oder eines Hämatoms an der Punktionsstelle erhöht.

### Aufklärung Injektion von Gelenken

Hier wird zusätzlich über das zu infiltrierende Medikament aufgeklärt. Bei geplanter **Infiltration von Medikamenten**

Infiltration von Medikamenten

in ein Gelenk muss im Rahmen der Vorbereitung auch die Frage nach einer evtl. vorliegenden **Medikamentenallergie**

Medikamentenallergie

gestellt werden.

Es sollte aufgeklärt werden über:

- obligatorisch: bakterielle Gelenkinfektion. In der Literatur werden Wahrscheinlichkeiten von ca. 0,04–0,08 % angegeben, dies entspricht 4 bis 8 Gelenkinfektionen auf 10.000 durchgeführte Punktionen [26, 27, 30];
- allergische Reaktionen bzw. Unverträglichkeitsreaktionen. Diese können durch ein eingespritztes Medikament, das Desinfektionsmittel oder auch durch Latex verursacht werden;
- systemische Arzneimittelreaktionen (z. B. bei Verwendung von Kortison-haltigen Medikamenten);
- Lokalreaktionen, die klinische Ähnlichkeit zum Infekt zeigen, davon aber klar abgegrenzt werden müssen. Die Symptomatik ist milder und legt sich meist innerhalb von 48 h;
- die empfohlene Schonung für mindestens 24 h nach der Punktion;
- Gefäßverletzungen. Das Spektrum reicht von kleineren Einblutungen bis hin zum Hämarthros und ist v. a. bei Patienten unter gerinnungswirksamen Substanzen zu bedenken;
- die Entwicklung einer sympathischen Reflexdystrophie der punktierten Region (vormals Morbus Sudeck);
- Knocheninfektionen, Verlust der Extremität, Sepsis mit potenziell tödlichem Ausgang (äußerst selten);
- Haut‑, Weichteil- oder Nervenschädigungen. Je nach geschädigter Struktur kann es zu Vernarbungen, Hämatomen, Abszessen, Schmerzen, Missempfindungen oder sogar Bewegungsstörungen kommen.

## Vorbereitung der Punktion

Die Vorbereitung einer Punktion richtet sich danach, welches Gelenk punktiert werden soll. So sollte z. B. bei der Punktion von Hüft- oder Handgelenken ein **Bildwandler**

Bildwandler

zu Hilfe genommen werden, um die sichere Punktion des Gelenks zu gewährleisten und zusätzlich eine radiologische Dokumentation der Punktion anzufertigen. Weiterhin können das Ertasten und Einzeichnen von **anatomischen Landmarken**

anatomischen Landmarken

eine große Hilfe sein. Die eingangs erwähnte Technik der Markierung der Einstichstelle mit dem Fingernagel hinterlässt eine auch nach dem Waschen sichtbare Delle bzw. Rötung. Im Zuge der Vorbereitung sollte der Patient je nach zu punktierendem Gelenk korrekt gelagert werden.

Bei der Gelenkpunktion hat das **aseptische Vorgehen**

aseptische Vorgehen

höchste Priorität. Das für die Intervention benötigte Instrumentarium, wird möglichst auf einem steril abgedeckten Tisch bereitgehalten. Hierbei hat sich eine **unsterile Assistenz**

unsterile Assistenz

bewährt, die unter Einhaltung der Asepsis die benötigten Utensilien zureicht.

Zur weiteren Asepsis sollte die Punktion mit Bedeckung der behaarten Kopfhaut und einem Mund-Nasen-Schutz erfolgen [3, 4]. Nach erfolgter chirurgischer Händedesinfektion werden sterile Operationshandschuhe benutzt. Die **Hautdesinfektion**

Hautdesinfektion

des zu punktierenden Gelenks sollte großflächig und mindestens 3‑mal erfolgen [3, 4]. Nach Abwarten der **Einwirkzeit**

Einwirkzeit

des jeweiligen Desinfektionsmittels kann mit dem Abdecken mittels steriler Operationstücher begonnen werden, meist reichen Lochtücher.

## Instrumentarium

**Figure 2:**
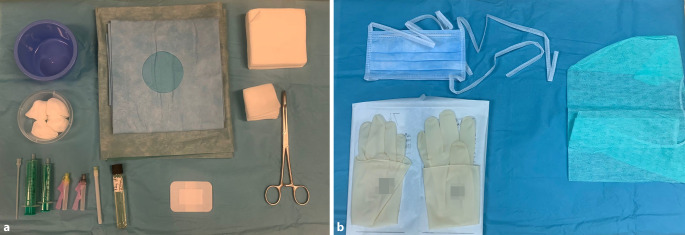

**Figure 3:**
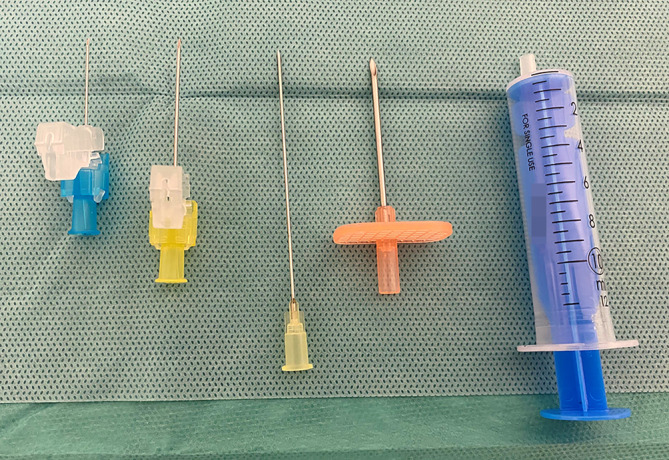

Vor der Punktion sollten alle Utensilien griffbereit vorbereitet sein. Das Instrumentarium für die Punktion eines Gelenks richtet sich nicht nur nach dem Gelenk, sondern auch nach der Intention, mit der das Gelenk punktiert wird. Auch die erwartete Konsistenz des Punktats hat auf das gewählte Instrumentarium Einfluss. Zur **optimalen Grundausstattung**

optimale Grundausstattung

für eine Gelenkpunktion gehören (Abb. 2):

- Mund-/Nasenschutz (Operationsmaske), Haube und sterile Handschuhe,
- Kornzange (fakultativ), Tupfer,
- Desinfektionsmittel (lokale Hygienebestimmungen sind zu beachten!),
- Lokalanästhetikum (fakultativ),
- Abdecktücher, z. B. Lochtuch mit oder ohne Klebekante,
- Punktionskanüle, Nierenschale/Gefäß,
- sterile Spritze (5ml, 10ml oder 20 ml)
- sterile Nadel in Abhängigkeit von Gelenk und Indikation (siehe Abb. 3)
- Proberöhrchen zum Versand für weitere Aufarbeitung,
- Verbandsmaterial, Pflaster,
- elastische Bandagen (von distal nach proximal über die Punktionsstelle; nicht praktikabel bei Punktion des Schulter‑, Hüft- und Iliosakralgelenks),
- Röntgenschutz für Patient und Personal sowie Beachtung der Strahlenhygiene bei Bildwandler-gestützer Punktion.

## Lagerung der Extremität

Der Patient sollte entspannt liegen oder sitzen. Gelegentlich ist es hilfreich, das Gelenk speziell zu lagern wie beim Abpunktieren eines schmerzhaften Gelenkergusses im Kniegelenk. Eine Rolle, die unter der Kniekehle platziert wird, schafft eine **entspannte Situation**

entspannte Situation

für den Eingriff und verkleinert bzw. entleert den dorsalen Gelenkraum. Am Kniegelenk kann durch eine geringe Lateralisierung der Patella mit dem Zeigefinger und durch manuellen Druck auf den Rezessus suprapatellaris der Erguss in den Recessus lateralis verlagert werden, wodurch der obere, laterale Recessus mit der Nadel leichter erreichbar wird und eine Aspiration leichter und sicherer gelingt (siehe Abb. 9a).

## Festlegung der Punktionsstelle mit und ohne Bildgebung

Die Punktion größerer und gut zugänglicher Gelenke (z. B. Knie, Sprunggelenk) erfolgt meist landmarkengestützt [31].

Bei schlechter zugänglichen Gelenken (insbesondere Hüfte, Schulter) wird die Punktion ultraschallgestützt erleichtert [31]. Ein klarer Vorteil der **Sonografie**

Sonografie

ist die einfache Handhabung durch immer kleiner werdende Geräte, Wiederholbarkeit, exakte Bestimmung der Eindringtiefe (für die Wahl der Nadellänge von Vorteil) und die fehlende Strahlenbelastung. Es hat sich gezeigt, dass im Vergleich zum landmarkengestützten Verfahren die ultraschallgeführte Punktion schmerzärmer und mit besserem Punktionserfolg bzw. größerer Punktionsmenge durchgeführt werden kann [10, 31, 32]. Um schmerzhafte Periostkontakte durch mehrfaches Sondieren und Aufsuchen des Gelenkspalts zu verhindern, kann die Punktion unter Röntgenbildwandler- oder Sonografiekontrolle erfolgen [33, 34].

**Bilderwandler-gestütze Punktionen** sind bei einigen Gelenken sinnvoll. Im Vergleich Bildwandler und Sonografie gab es eine höhere Treffsicherheit für den Bildwandler bei der Punktion des Iliosakralgelenks (ISG), wobei erwähnt werden muss, dass die korrekte Punktionsstelle in 2 Ebenen dargestellt werden muss [35, 36]. Bei korrekter Durchführung und entsprechender Expertise sind allerdings sehr gute Ergebnisse mit der Ultraschall-gezielten Methode erreichbar, wobei spezielle Punktionsaufsätze für die Ultraschallköpfe (Abb. 1) unter Berücksichtigung der Eindringtiefe und des Winkels die Treffsicherheit erhöhen [37].

**Bildgebungsgestützte Punktionen**

Bildgebungsgestützte Punktionen

wie CT- oder MRT-gesteuerte Punktionen sind möglich, werden in dieser Arbeit aber nicht erläutert, da sie Spezialindikationen vorbehalten sind [38]. Um das Risiko einer Punctio sicca (Punktion ohne Aspirat) zu reduzieren, können in Ausnahmefällen auch Computertomographie-gezielte Punktionen beispielsweise an der Hüfte erfolgen. Die MRT spielt in der Punktion von Gelenken eine untergeordnete Rolle und findet eher in der Radiologie Anwendung.

Die **Punktionslokalisation**

Punktionslokalisation

gemäß den anatomischen Landmarken auch nach endoprothetischer Versorgung bringt gute Treffsicherheit [8]. Allerdings können wiederum bei adipösen Patienten, lokalen Schwellungen oder Tumoren die Bedingungen erschwert sein und bildgebende Verfahren notwendig werden.

## Punktionsnadeln

Die **Nadellänge**

Nadellänge

richtet sich nach der zu erwarteten Eindringtiefe, die an bestimmten Stellen wie beispielsweise dem Hüftgelenk bzw. bei korpulenteren Patienten länger sein muss. Bei Unklarheit der verwendeten Nadellänge ist es sinnvoll, eine längere Nadel zu verwenden, um mehrere Punktionsversuche – mit Wechsel der Kanüle – zu vermeiden. Für die Punktion von Hüft- und Schultergelenk werden in der Regel lange Kanülen benötigt. Bei den hier nicht abgehandelten kleineren Gelenken sind kürzere Nadeln vorzuziehen, da diese eine bessere Haptik für den Untersucher vermitteln.

Die **Nadeldurchmesser**

Nadeldurchmesser

(Gauge) und Nadellänge (Inch) werden gewählt nach der Größe des Gelenks, dem Weichteilmantel und der zu erwartenden Viskosität des Punktats. Die Punktion eines länger bestehenden Hämarthros wird nur mit dickerer Nadel oder überhaupt erst über einen Arthroskopietrokar möglich sein, ebenso wird die Injektionsflüssigkeit die Nadelgröße (z. B. zähflüssige Hyaluronsäurepräparate) beeinflussen. Zusammenfassend gilt, dass kleinvolumige Nadeln weniger Schmerzen verursachen. Zur Nadelwahl gilt es, den richtigen Kompromiss zu finden.

### Merke

So zart wie möglich, so groß wie nötig.

Bei Knochen- bzw. Knorpelkontakt der Nadel wird die Punktionskanüle zurückgezogen und anatomiegerecht vorsichtig erneut vorgeschoben.

Bei Verwendung einer Nadel mit größerem Durchmesser und einer großvolumigen Spritze (stärkere Saugkraft) lassen sich auch **dickflüssige Gelenkflüssigkeiten**

dickflüssige Gelenkflüssigkeiten

aspirieren. Ist ein zähflüssiges Punktat zu erwarten, wie bei nicht frischem Hämarthros, wird nach vorheriger örtlicher Betäubung und eventueller Stichinzision eine großlumige Punktionsnadel (z. B. DIN EN ISO 9626 [Strauss]) verwendet. Die Inzision soll das Einbringen eines Hautstanzzylinders durch die großlumige Nadel verhindern. Durch die große Öffnung der Kanüle kann auch dickflüssiges Punktat suffizient abgesaugt werden. Die Hautinzision wird danach mittels Pflasterstrip oder Einzelknopfnaht verschlossen.

Für den sterilen Verschluss der Punktionsstelle reicht in der Regel ein steriles Pflaster, das für einen Tag belassen werden sollte.

## Lokalanästhesie

Eine (Lokal‑)Anästhesie sollte nach individueller Situation verabreicht werden. Topische **Lidocain**

Lidocain

-Salben und -Gele können v. a. bei Kindern oder ängstlich schmerzbehafteten Patienten sinnvoll sein [39] – auch vor **Setzen einer Quaddel**

Setzen einer Quaddel.

Nach der Vorbereitung der Haut, dem Abdecken und der Lokalisation der Einstichstelle für die Nadel wird an der Eintrittsstelle zunächst eine Quaddel mit dem Anästhetikum (z. B. Lidocain 1 %) [26, 27] gesetzt. Nach Einsetzen der Wirkung werden weitere 2 bis max. 5 ml Lokalanästhetikum fächerförmig ins subkutane Gewebe injiziert [26, 27]. Bei der Verwendung einer Lokalanästhesie sollte bedacht werden, dass der Wirkungseintritt vom lokalen pH-Wert abhängt und v. a. in entzündetem Gewebe mit niedrigem pH-Wert verzögert oder die Wirkung abgeschwächt sein kann [38].

Bei lokaler Betäubung sollte bei **rheumatologischer Fragestellung**

rheumatologischer Fragestellung

darauf geachtet werden, dass kein Anästhetikum in die Gelenkhöhle gespritzt wird, da die Genauigkeit der Zellzahlergebnisse verfälscht werden kann.

## Untersuchung der gewonnenen Gelenkflüssigkeit

Soll das Punktat einer Begutachtung zugeführt werden, hat es sich bewährt, ein steriles Gefäß bereitzuhalten, in dem bereits eine makroskopische erste Begutachtung möglich ist. So können bei blutigem Punktat auf der Oberfläche schwimmende Fettaugen (= **Liphämarthros**

Liphämarthros)

erkannt werden, die Einfluss auf die weitere Diagnostik haben.

Labortests für Synovialflüssigkeit beinhalten bakteriologische Untersuchungen wie die Gram-Färbung und Kultur oder die mikroskopische bzw. laborchemische Aufarbeitung hinsichtlich Zellzahl, Kristalle, pH-Wert, Lactat oder Urat.

## Punktionstechniken der großen Gelenke

**Figure 4:**
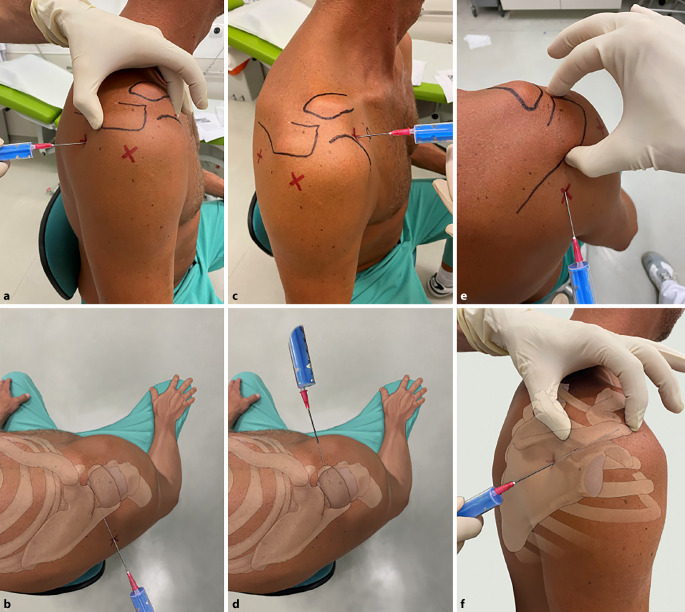

**Figure 5:**
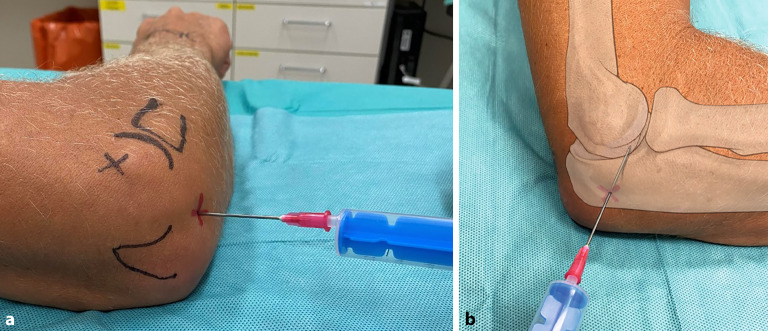

**Figure 6:**
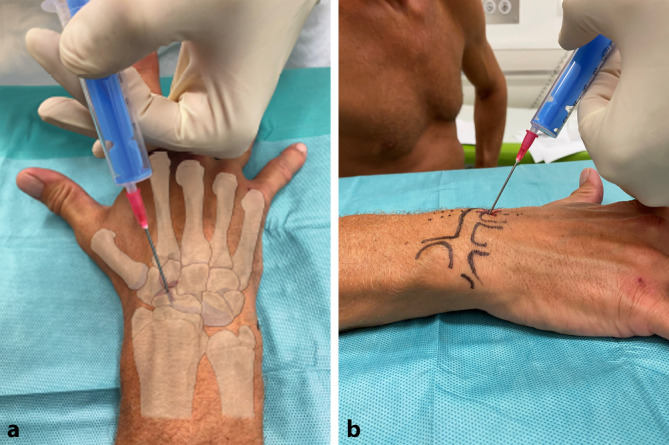

**Figure 7:**
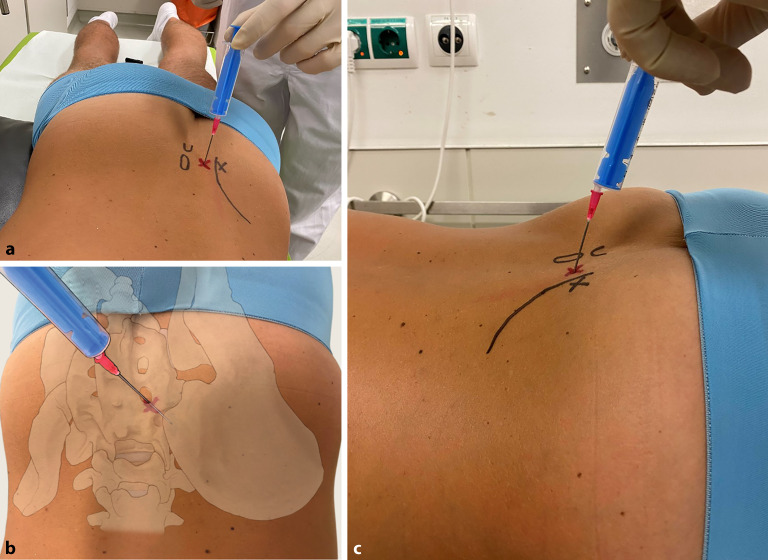

**Figure 8:**
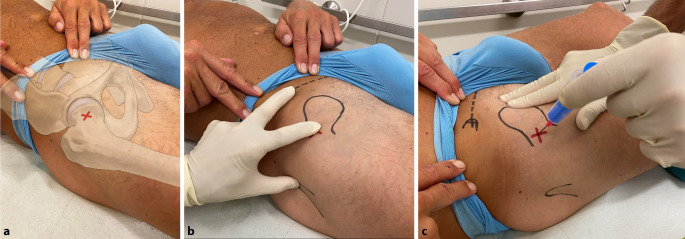

Auf die Verwendung einer sterilen Abdeckung bzw. eines Lochtuches wurde in den Abbildungen aus Gründen der besseren Darstellung verzichtet (Abb. 4, 5, 6, 7, 8, 9 und 10).

### Schultergelenk

#### Indikationen

Siehe allgemeine Indikationen.

#### Lagerung

Der Patient sitzt aufrecht auf der Untersuchungsliege. Der betroffene Arm ist adduziert, Hand und Unterarm ruhen auf dem Unterbauch, auf kontralateralem Unterarm und Hand oder am gleichseitigen Oberschenkel (Abb. 4b). Die Muskulatur ist entspannt.

#### Landmarken/Zugang

(Abb. 4)

**Orientierungspunkte**

Orientierungspunkte

können vor der Desinfektion mit einem Marker gekennzeichnet werden. Folgende Schritte haben sich bewährt: Palpation und Markierung des Korakoids in der Kerbe, in der sich Schulterdach (Akromion) und Schlüsselbein treffen, anterolaterale und posterolaterale Ecke des Akromions. Es resultiert ein gleichseitiges Dreieck. Markierung der lateralen Kante des Akromions sowie von Klavikula und Spina scapulae. Je nach Weichteilmantel und Habitus lassen sich die Markierungspunkte teilweise leichter von anterior, meist jedoch gut von posterior palpieren.

- Dorsaler Zugang (Abb. 4a, b)
  Das Glenohumeralgelenk kann am einfachsten beim sitzenden Patienten von dorsal punktiert werden. Der Eintrittspunkt liegt 2 Querfinger (ca. 2–3 cm) inferior und medial der posterolateralen Begrenzung des Akromions. Die Punktion erfolgt im dorsalen Soft-Spot mit Zielrichtung auf das Korakoid [32].
- Ventraler Zugang (Abb. 4c, d)
  Beim ventralen oder anterioren Zugang wird direkt lateral vom Orientierungspunkt Korakoid zwischen Humeruskopf und lateraler Klavikula punktiert. Zielen Sie direkt nach posterior und leicht superior Richtung Fossa glenoidalis. So wird verhindert, dass neurovaskuläre Strukturen verletzt werden.

##### Merke

Keine Punktion medial des Korakoids!

- Der Subakromialraum (Abb. 4e, f) kann sowohl von dorsal (wie das Glenohumeralgelenk) als auch von lateral punktiert werden. Der laterale Eintrittspunkt liegt 2 cm unter der Mitte des lateralen Akromions. Der Raum zwischen Akromionunterrand und Humeruskopf wird palpiert. Der Patient wird gebeten, den Arm wirklich locker hängen zu lassen, um die akromiohumerale Distanz möglichst zu erweitern.
- Das Akromioklavikulargelenk wird von kranial in orthogonaler Richtung punktiert, wobei die Verwendung eines Bildwandlers empfehlenswert ist.

### Ellenbogengelenk

#### Indikationen

Siehe allgemeine Indikationen.

#### Hinweise – Vermeidung von Komplikationen

Siehe Komplikationen.

Bevor das Gelenk punktiert wird, sollten andere Entitäten ausgeschlossen werden: u. a. Bursitis olecrani, Epicondylitis humeri (Tennisellenbogen), Kontusion und dermatologische Entzündung.

#### Lagerung

Patient sitzend auf Liege, Arm gelagert in Pronation auf Handtisch, den Ellenbogen ca. 90° gebeugt.

#### Landmarken/Zugang

(Abb. 5)

Palpation und Markierung des lateralen Epikondylus bei Beugung des Ellenbogens, gefolgt von der Palpation des Radiusköpfchens, welche durch Pro- und Supination erleichtert wird. Nach Lokalisation der Olekranonspitze ergibt sich in etwa ein gleichseitiges Dreieck zwischen den drei anatomischen Punkten.

Der **Eintrittspunkt**

Eintrittspunkt

liegt in der Mitte des Dreiecks in einem Soft-Spot (Abb. 5a, b). Die Nadel wird senkrecht zur Hautoberfläche gestochen.

- Die Ellenbogenpunktion erfolgt in 45–90° Flexion und in Pronation. Dadurch erweitert sich der Gelenkspalt im Humeroradialgelenk [11].
- Die Punktionsstelle liegt distal des lateralen Epikondylus und anterior des Olekranons im Anconeusdreieck.
- Die geübte Praxis der Punktion bei Radiusköpfchenfrakturen zeigt keine genügende Evidenz [40], kann jedoch zur Schmerzlinderung beitragen.

### Handgelenk

#### Indikationen

Siehe allgemeine Indikationen, spezielle Indikation wäre die **Probeinfiltration**

Probeinfiltration

bezüglich Schmerzausschaltung für spätere Arthrodese.

#### Lagerung

Hand und Unterarm liegen auf einer Auflage, z. B. fahrbarer Handtisch. Das Handgelenk ist proniert, etwas gebeugt und in leichter Ulnardeviation. Distraktion manuell durch den Untersucher oder mittels Mädchenfänger kann hilfreich sein, ebenso die Verwendung eines Bildwandlers.

#### Landmarken/Zugang

(Abb. 6)

- In Pronationsstellung kann der Gelenkspalt über dem distalen Radius palpiert werden. Die Punktionsstelle liegt am dorsalen Handgelenk distal des tastbaren Lister-Tuberkels zwischen der langen Strecksehne des Daumens (EPL) und den Strecksehnen der Langfinger (EDC) [6, 7]. Hier findet sich ein Soft-Spot entsprechend dem dorsalen Handgelenkarthroskopieportal 3–4 bzw. zwischen dem 3. und 4. Strecksehnenfach.
- Falls die senkrecht eingeführte Nadel knöchernen Kontakt hat, wird sie subkutan zurückgezogen und anschließend neu vorgeschoben, ggf. Bildwandler verwenden.
- Die Gelenksflächenneigung in zwei Ebenen muss berücksichtigt werden. Die Gelenkfläche fällt nach ulnar und nach volar ab, entsprechend wird die zu Beginn senkrecht eingeführte Nadel ausgerichtet.

### Iliosakralgelenk

#### Indikationen

Siehe allgemeine Indikationen; spezielle Indikation ist die Infiltration von Kortikosteroiden mit Lokalanästhetikum bei rheumatischer Erkrankung (z. B. Morbus Bechterew).

#### Lagerung

Der Patient liegt auf dem Bauch, Verwendung einer strahlendurchlässigen Untersuchungsliege für die Bildwandler-gestützte Durchführung.

#### Landmarken/Zugang

(Abb. 7)

- Aufgrund der anatomischen Gegebenheiten sowie der Nahebeziehung des ISGs zum N. ischiadicus ist eine bildgebungsgestützte Punktion des Gelenks zu empfehlen.
- Die unteren zwei Drittel des Gelenks sind mit Synovia (d. h. für die Punktion geeignet), das obere Drittel ist mit derbem Bindegewebe als Amphiarthose ausgestattet.
- Der Patient wird in leicht aufgekippter Bauchlage (Polster unter der kontralateralen Beckenhälfte!) gelagert, der Bildwandler geschwenkt, um das Gelenk anteroposterior einzusehen.
- Die Nadel wird in dieser Lage vertikal zum Boden, ggf. mit leicht kranialer Stichführung (20–25°) eingebracht (Abb. 7) und die Lage der Spitze mittels Bildgebung kontrolliert [35].

### Hüftgelenk

#### Indikationen

Siehe allgemeine Indikationen; spezielle Indikation wäre die Punktion zum Ausschluss eines Protheseninfektes.

#### Lagerung

Patient liegt mit dem Rücken auf einer strahlendurchlässigen Liege.

#### Landmarken/Zugang

(Abb. 8)

- Wegen der Weichteildeckung des Hüftgelenks sowie der anspruchsvollen Punktionstechnik empfiehlt sich die Bildwandler-gezielte Punktion der Hüfte.
- Der Patient wird in Rückenlage gelagert, und der femorale Gefäß-Nerven-Strang (Abb. 8c) sowie das Ligamentum inguinale werden getastet. Der Eintrittspunkt liegt lateral der A. femoralis und 2 cm unter dem Ligamentum inguinale (alternative Höhenbestimmung über die Spitze des Trochanter major). Als zusätzliche Orientierung kann mit 3 Fingern ein gleichschenkeliges Dreieck zwischen der Spina iliaca anterior superior, dem Trochanter major und dem Punktionspunkt gelegt werden (Abb. 8a, b).
- Die korrekte Lage der Nadel im Gelenk bzw. an der Prothese wird mittels Bildwandler bestätigt und dokumentiert.

### Kniegelenk

#### Hinweise – Vermeidung von Komplikationen

Zur Abklärung, ob intra- oder extraartikuläre Ergussbildung vorliegt, kann die Ultraschalluntersuchung helfen. Bei extraartikulärer Ergussbildung ist die Punktion des Kniegelenks wegen Keimverschleppung kontraindiziert.

#### Lagerung

**Figure 9:**
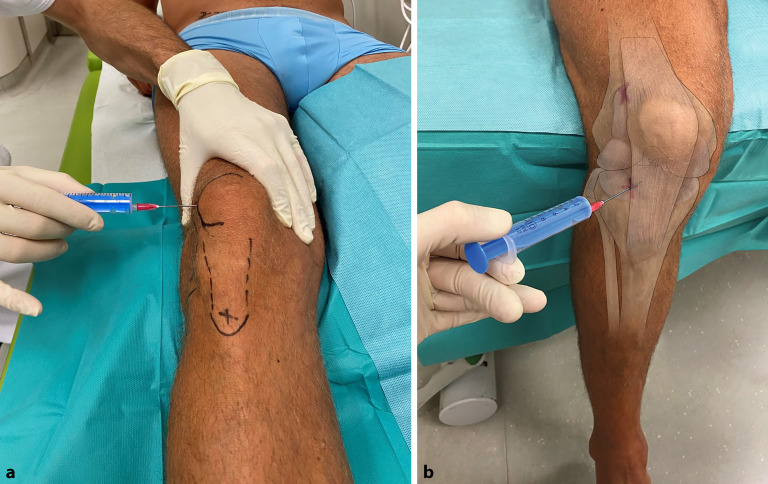
- Anterolateraler Zugang
  Punktion auf Kniegelenksspalthöhe, knapp lateral des meist gut tastbaren Ligamentum patellae Richtung Intercondylärregion. entsprechend dem Soft-Spot des anterolateralen Arthroskopieportals (Abb. 9b).

- Patient liegt entspannt in Rückenlage, Knie extendiert oder Rolle (presst den dorsalen Gelenkraum aus) unter der Kniekehle/dem Kniegelenk bei größeren Gelenkergüssen (leichte Beugung wird angenehmer und entspannter empfunden) (Abb. 9a).
- Alternativ sitzt der Patient mit herabhängendem Unterschenkel (ca. 90° Flexion) auf der Untersuchungsliege (Abb. 9b), die Punktion erfolgt im Dreieck von Tibiavorderkante, Femurkondylus und Patellarsehne (Soft-Spot) in Richtung Kniegelenkzentrum – entsprechend dem anterolateralen paraligamentären Arthroskopieportal [41].

#### Landmarken/Zugang

Die Punktion kann entweder von **lateral oder medial**

lateral oder medial

erfolgen, wobei hier die bevorzugte Vorgehensweise von lateral dargestellt wird. Die Genauigkeit der Punktion in das Kniegelenk kann durch den Einsatz von Ultraschall verbessert werden [42].

Der geplante Punktionsort kann im Bedarfsfall mit einem Lokalanästhetikum gequaddelt werden, natürlich ohne den Gelenkraum zu treffen.

- Superolateraler Zugang
  Die Punktionsstelle liegt etwa 1 cm oberhalb und 1 cm lateral des oberen lateralen Aspekts der Kniescheibe ([42]; Abb. 9a). Hilfreich ist auch ein die Kniegelenkscheibe umgreifender Druck auf den Recessus suprapatellaris, der Zeigefinger kann dabei die Patella leicht lateralisieren. Dadurch kommt es zu einer Verlagerung des Ergusses in den lateralen oberen Recessusanteil.

### Oberes Sprunggelenk

#### Indikationen

Siehe allgemeine Indikationen; spezielle Indikation wäre die Probebehandlung mittels Lokalanästhetikum zur Schmerzausschaltung bezüglich **nachfolgender Arthrodese**

nachfolgender Arthrodese

(speziell auch im unteren Sprunggelenk).

#### Hinweise – Vermeidung von Komplikationen

Siehe Komplikationen; gerne verwenden wir auch vor dem sterilen Waschen ein **desinfizierendes Fußbad**

desinfizierendes Fußbad.

Aufgrund der regionsbedingten Ödemneigung (Infektgefahr) ist besondere Sorgfalt auf die aseptische Technik zu legen.

##### Lagerung.

Der Patient sitzt mit herabhängendem Bein auf der Untersuchungsliege oder liegt am Rücken.

#### Landmarken/Zugang

Anatomische Landmarken für die Punktion des oberen Sprunggelenkes (OSG) sind die knöchernen Strukturen des Innen- und Außenknöchels sowie die Sehnen Extensor digitorum longus (EDL), Extensor hallucis longus (EHL) und Tibialis anterior (TA).

##### Zugänge zum oberen Sprunggelenk.

(Abb. 10)

**Figure 10:**
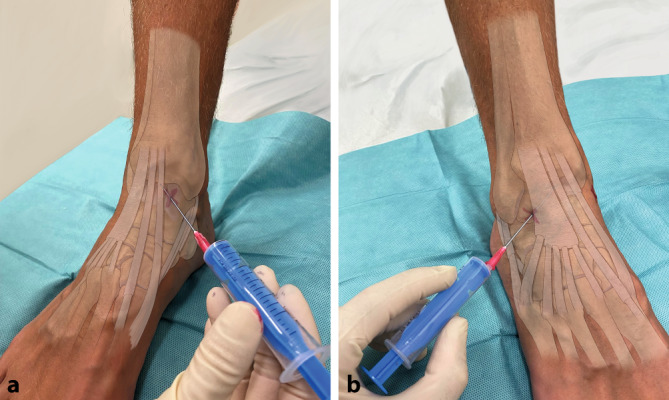

- Anteromedialer Zugang
  Die Punktion erfolgt in leichter Plantarflexion des Fußes. Der Soft-Spot liegt zwischen Innenknöchel und Tibialis anterior Sehne auf Höhe des Gelenkspaltes (Abb. 10a; [41]). Hierbei ist eine Verletzung der Dorsalis-pedis-Gefäße und der tiefen Peroneusnervenäste zu vermeiden, die sich unmittelbar unterhalb und lateral der EHL-Sehne finden. Bei Fußstreckung gegen Widerstand oder bei aktiver Dorsal- und Plantarflexion ist der Soft-Spot medial der EHL- und TA-Sehne leichter zu identifizieren.
- Anterolateraler Zugang
  Zwischen der Sehne des Extensor digitorum communis (EDC) und dem Außenknöchel befindet sich der Soft-Spot für den anterolateralen Zugang zum oberen Sprunggelenk (Abb. 10b). Bei diesem Zugang ist auf die Endäste des N. peroneus superficialis (Hautnerven) zu achten, die in Folge einer direkten Punktion verletzt werden könnten.

Die Nadel wird auf Höhe der Gelenklinie tangential zur Krümmung der Talusrolle geführt, eine übermäßig Auslenkung der Nadel aus der Gelenklinie ist zu vermeiden [6, 7].

## Nachbehandlung

- Sterile (Pflaster‑)Verbandanlage postinterventionell
- Abhängig von Punktionslokalisation und Gerinnungsstatus ist eine ambulante Nachbeobachtung von 15–20 Minuten zu erwägen.
- In speziellen Fällen (z. B. Komplikation durch Punktion einer Vene oder Arterie) sind eine verlängerte Beobachtungsphase, Kryotherapie und Kompressionsverbandanlage angezeigt [11, 41].
- Ein elastischer Kompressionsverband sollte immer von distal (Mittelhand/Mittelfuß) bis über die Punktionsstelle geführt werden – sonst besteht Stauungsgefahr mit der Möglichkeit der Thrombosebildung (nicht angebracht bei Schulter‑, Hüft- und ISG-Punktionen – alternativ kann bei Hüftpunktionen ein handelsüblicher leichter Kompressionsverband für die Hüftregion, wie er in vielen Häusern postoperativ nach Hüftprothesenoperationen verwendet wird, nach vorheriger Bandagierung der unteren Extremität angewandt werden).

## Fehler/Gefahren/Komplikationen

- Kaum Blutungsgefahr bei Einnahme von oralen Antikoagulanzien [1, 9, 25]
- Blutungsrisiko minimiert, wenn Punktion des Gelenks korrekt durchgeführt
- Schwellungen und Schmerzen nach Gelenkpunktion sehr selten (< 0,01 %) [13]
- Risiko für iatrogene Gelenkinfektionen zwischen 0,04 und 0,08 % [26, 27, 30]
- Anteil falsch positiver Punktate bei Gelenkaspiration gegen 0 % wenn korrekt durchgeführt [43]
- Geringer Erfahrungswert erhöht Komplikationsgefahr [13]
- Falsch negative Punctio sicca bei geringer Nadeldicke und viskösem/flockigem Gelenkerguss (z. B. Eiter, geronnenem Blut bei Hämarthros) [6, 7]. Tipp: Drehen des Schliffes der Nadel
- Forcierte Kanüleneinbringung kann zu Knorpelabrasionen und -schaden führen. Der Vorschub ist zu stoppen, sobald Flüssigkeit über die Nadel austritt, womit Knorpelschäden vermieden werden.

## Punktataufarbeitung, Ergebnisinterpretation

Die **Analyse**

Analyse

der entnommenen Gelenkflüssigkeit kann bei der Aufklärung der Ätiologie helfen [41].

**Figure 11:**
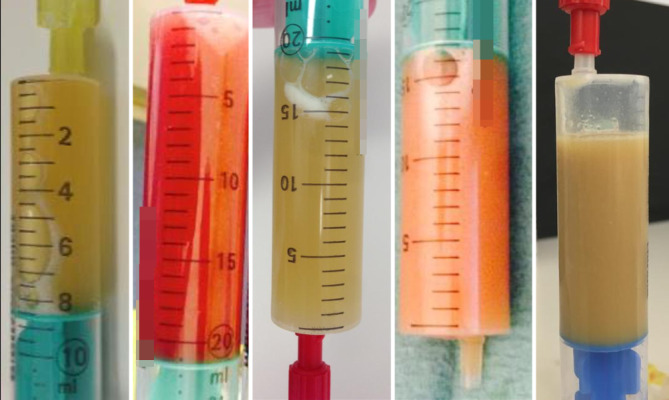

- Erscheinung des Punktats (Abb. 11)
  - Je schwerer die Gelenkentzündung, desto höher die Zellzahl in der Synovialflüssigkeit und desto trüber ihr Aussehen [6, 7]
  - Putrides Punktat als Zeichen eines Pyarthros, flockiges Punktat als Zeichen einer Kristallarthropathie/Chondrokalzinose [6, 7]
  - Blutiges Punktat als Zeichen eines Hämarthros. Superinfektion dennoch möglich.
- Synoviaanalyse
  - Zellzahl – Leukozyten über 3000/μl oder > 80 % Granulozyten sind bei der Punktion eines Gelenkersatzes beweisend für einen Protheseninfekt [44]
    Derzeit sind keine derartigen Grenzwerte für Nativgelenke beschrieben
  - Positive Kristallanalyse beweisend für das Bestehen einer Gicht (Uratkristalle) [45] oder Pseudogicht (Calciumpyrophosphat-Dihydrat[CPPD]-Kristalle) [46]. Eine Superinfektion ist damit dennoch nicht ausgeschlossen.
- Mikrobiologische Aufarbeitung
  - Das Punktat wird standardmäßig in BHI (Brain-Heart-Infusion), ähnlich einer Agarlösung als Nährboden, alternativ in Blutkulturfläschchen zur Keimbebrütung versandt (10 bis 14 Tage).
  - Eine positive Kultur weist auf eine Gelenksinfektion hin.
  - Zwei positive Kulturen desselben Keims (mit Antibiogramm) beweisen eine Gelenkinfektion. Bei hochvirulenten Organismen (Staphylococcus aureus oder Gram-negative Stäbchen) reicht ein einziger Nachweis.
  - In einer eigenen Untersuchungsserie wurden im Jahr 2020 im Rahmen einer Synoviaanalysestudie (*n* = 2176) am häufigsten die Keime *Staphylococcus aureus* (31,7 %) und *Staphylococcus epidermidis* (15,2 %) als Erreger nachgewiesen. Der Literaturvergleich zeigt ähnliche Ergebnisse [47].
  - Achtung: Unter laufender Antibiose kann das Punktat falsch negativ sein. Daher ist die Interpretation des Punktats immer ein Zusammenspiel von mehreren Ergebnissen.

## CME-Fragebogen

Welche anatomische Lagebeziehung beschreibt eine korrekte Punktionsstelle am Ellenbogen?

Lateraler Epikondylus – Radiusköpfchen – Olekranon

Trochlea – Radiusköpfchen – Olekranon

Medialer Epikondylus – Trochlea – Coronoid

Radiusköpfchen – Capitellum – Coronoid

Radiusköpfchen – lateraler Epikondylus – Coronoid

Die ausführliche Aufklärung über das Risiko einer Komplikation in Zusammenhang mit einer Gelenkpunktion ist ärztliche Pflicht. Vor allem Infektionen stellen eine gefährliche Komplikation dar. Wie hoch ist das Risiko einer bakteriellen Infektion trotz Beachtung aller Sterilitätskriterien?

4 bis 8 Infektionen bei 100 Punktionen

4 bis 8 Infektionen bei 1000 Punktionen

4 bis 8 Infektionen bei 10.000 Punktionen

4 bis 8 Infektionen bei 100.000 Punktionen

4 bis 8 Infektionen bei 1.000.000 Punktionen

Welche Aussage zur Punktion an der Schulter ist korrekt?

Das Akromioklavikulargelenk wird optimalerweise von dorsal punktiert.

Der ventrale Eintrittspunkt liegt medial des Korakoids.

Der dorsale Eintrittspunkt liegt 1–2 cm inferior und medial des posterolateralen Akromions.

Der Subakromialraum kann sowohl von dorsal wie auch medial punktiert werden.

Bei der Punktion von ventral zielt die Nadel in Richtung Korakoid.

Die sterile Punktion großer Gelenke stellt eine simple, risikoarme und minimalinvasive Technik dar. Welche der unten genannten Konstellationen stellt *keine* Indikation für eine Punktion dar?

Abpunktion von Reizerguss

Keimgewinnung bei Verdacht auf septische Arthritis

Hämatomentlastung nach Trauma

Medikamenteninjektion bei Arthrosebeschwerden

Kortikoidinjektion vor geplantem endoprothetischem Gelenkersatz

Ein 58-jähriger Patient kommt mit generalisierter Infektsymptomatik (Fieber 38,7 °C, Schüttelfrost) zu Ihnen in die Notfallambulanz. Als möglichen Infektfokus identifizieren Sie ein gerötetes, überwärmtes Kniegelenk mit Zeichen eines intraartikulären Ergusses. Sie suspizieren eine septische Arthritis und führen eine intraartikuläre Punktion durch. Wie punktieren Sie das Kniegelenk standardmäßig?

Patient steht, Knie leicht flektiert, Einstich superolateral der Patella

Patient in Rückenlage, Einstich anteromedial der Patellasehne

Patient sitzt mit 90° flektiertem Unterschenkel auf der Liege, Einstich superolateral der Patella bei Traktion derselben durch den Untersucher

Patient in Rückenlage, Einstich superolateral der Patella bei Medialisierung derselben durch den Untersucher

Patient in Rückenlage, Einstich superolateral der Patella bei Lateralisierung durch den Untersucher

Ein 58-jähriger Patient kommt mit generalisierter Infektsymptomatik (Fieber 38,7 °C, Schüttelfrost) zu Ihnen in die Notfallambulanz. Als möglichen Infektfokus identifizieren Sie ein gerötetes, überwärmtes Kniegelenk mit Zeichen eines intraartikulären Ergusses. Sie suspizieren eine septische Arthritis und führen eine intraartikuläre Punktion durch. Welche Erscheinung des Punktats passt zu Ihrer Verdachtsdiagnose septische Arthritis?

Klar, bernsteinfarben

Hellgelb, hohe Viskosität

Blutig mit Koageln

Trüb, niedrig viskös, grün-gräuliche Farbe

Gelb, mit vermehrt flockiger Beimengung

Ein 58-jähriger Patient kommt mit generalisierter Infektsymptomatik (Fieber 38,7 °C, Schüttelfrost) zu Ihnen in die Notfallambulanz. Als möglichen Infektfokus identifizieren Sie ein gerötetes, überwärmtes Kniegelenk mit Zeichen eines intraartikulären Ergusses. Sie suspizieren eine septische Arthritis und führen eine intraartikuläre Punktion durch. Welche Aussage zur Bestätigung der Verdachtsdiagnose septische Arthritis ist zutreffend?

Bei Bestätigung einer CPPD(Calciumpyrophosphat-Dihydrat)-Kristallarthropathie ist eine septische Arthritis ausgeschlossen.

Eine mikrobiologische Aufarbeitung ohne Keimnachweis schließt eine septische Arthritis aus.

Das gleichzeitige Bestehen einer Kristallarthropathie und einer bakteriellen Infektion ist möglich und erschwert den diagnostischen Prozess.

Drei Tage nach Bebrütung ist ein mikrobiologisches Endergebnis zu erwarten, ein späteres Keimwachstum deutet in erster Linie auf eine Kontamination hin.

Die Interpretation des mikrobiologischen Ergebnisses ist nach Ex-juvantibus-Antibiotikagabe unmöglich.

Bezüglich der Punktionskanüle zur sterilen Punktion großer Gelenke gilt folgender Grundsatz:

So lang wie möglich, maximal dünn

So dick wie möglich, damit sie ja nicht bricht

So stark wie möglich, damit sie sich ja nicht verbiegt

So klein wie möglich, so groß als nötig

So stumpf wie möglich, damit keine Schmerzen auftreten

Welche Aussage bezüglich einiger Punkte der Patientenaufklärung vor der Punktion eines Gelenkes ist richtig?

Nach Punktion eines großen Gelenkes sollte konsequent eine Ruhigstellung in einer Schiene o. Ä. erfolgen.

Wenn allgemein keine Allergien bekannt sind, erübrigt sich der Hinweis auf mögliche systemische Reaktionen.

Eine mögliche nachfolgende Gelenkinfektion kann unabhängig von der Indikation zu einer Knocheninfektion führen.

Bei Patienten mit Einnahme von gerinnungshemmenden Medikamenten ist die Punktion eines großen Gelenkes nicht möglich.

Lokale Reaktionen nach Injektion auf ein Medikament sind als ähnlich schwerwiegend wie die systemische Reaktion zu werten.

Der folgende Begriff bezüglich der sterilen Punktion großer Gelenke ist richtig:

Fettaugen auf dem Punktat zeugen von einer kräftigen Subkutis.

Als Hämarthros wird ein Reizerguss im Kniegelenk bezeichnet.

Ein Liphämarthros ist verdächtig auf eine Verletzung der subchondralen Knorpel‑/Knochengrenzlamelle.

Ein Reizerguss des Schultergelenkes ist durch Bakterien in der mikroskopischen Aufarbeitung des Punktates charakterisiert.

Kristalle in der histologischen Aufarbeitung des Punktates zeugen von einer iatrogenen Infektion.
